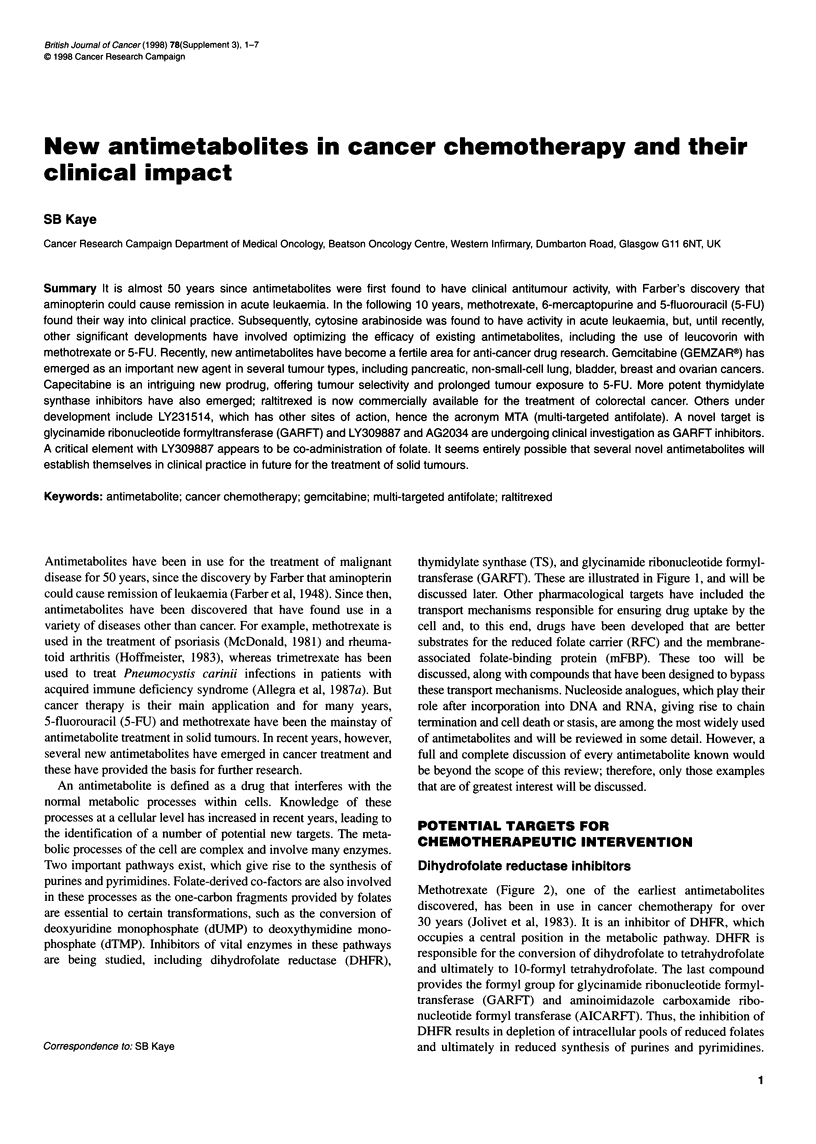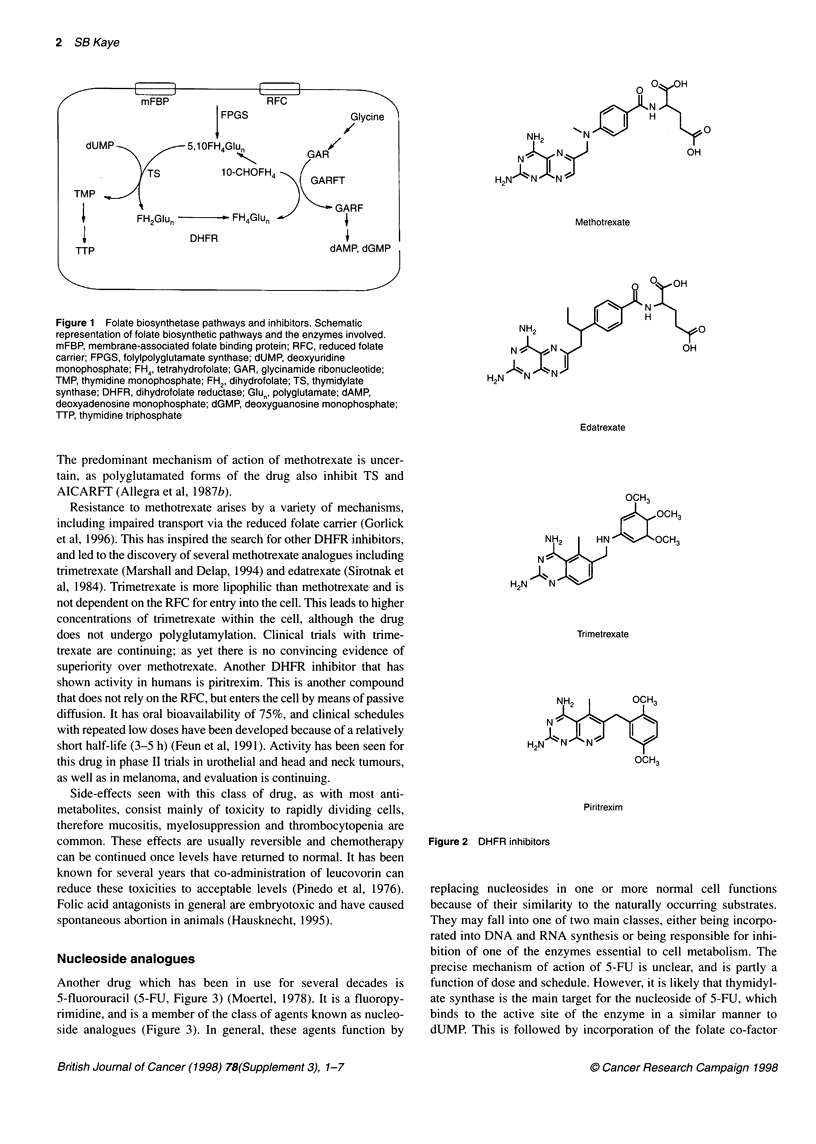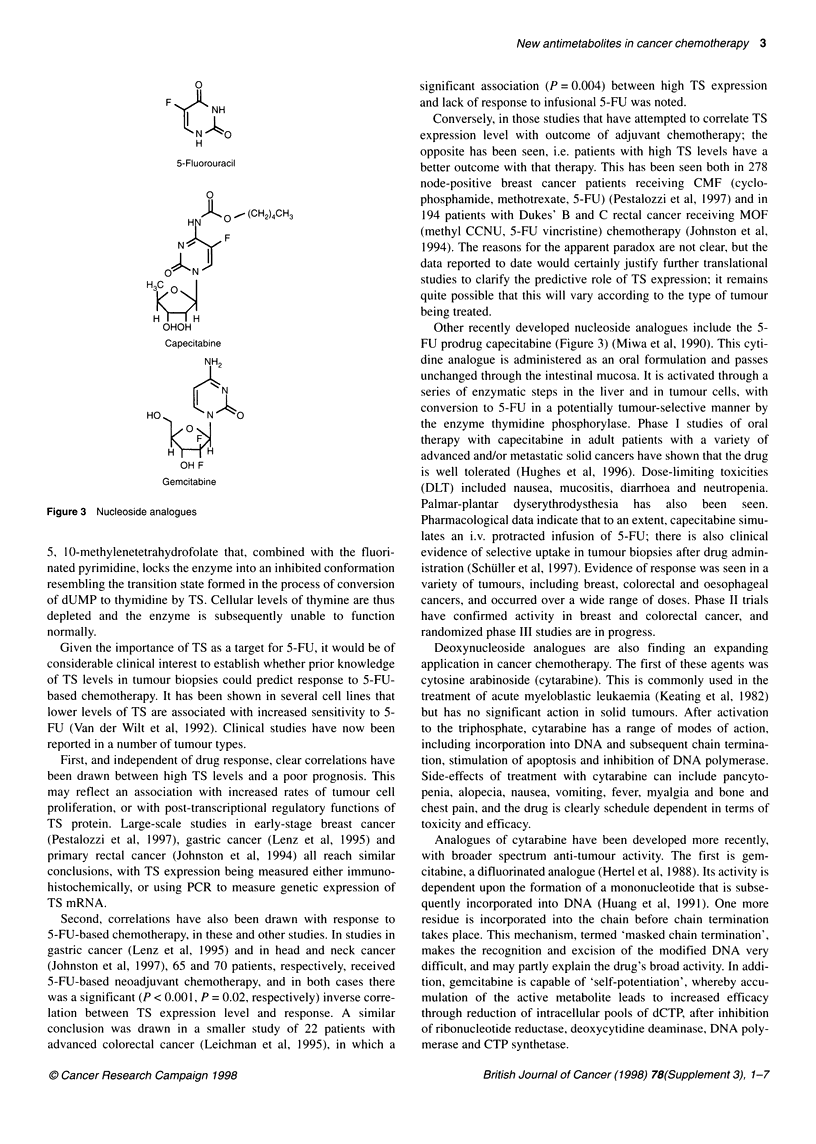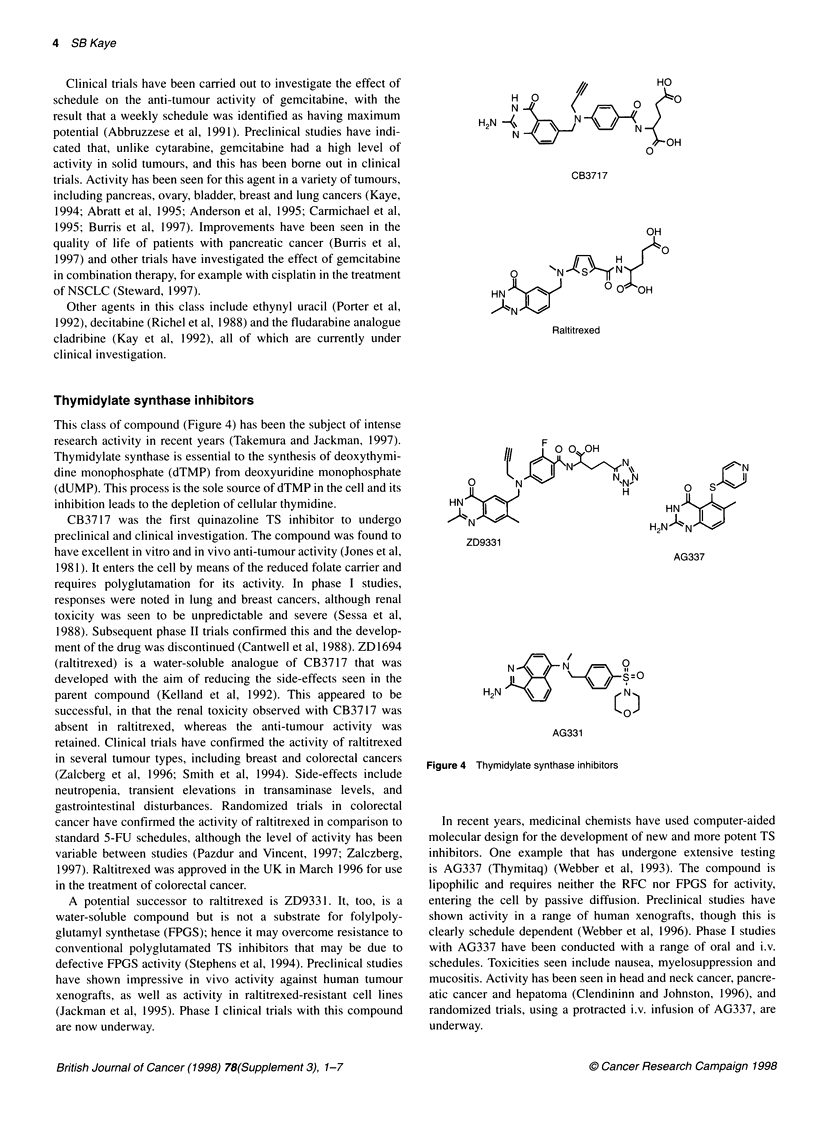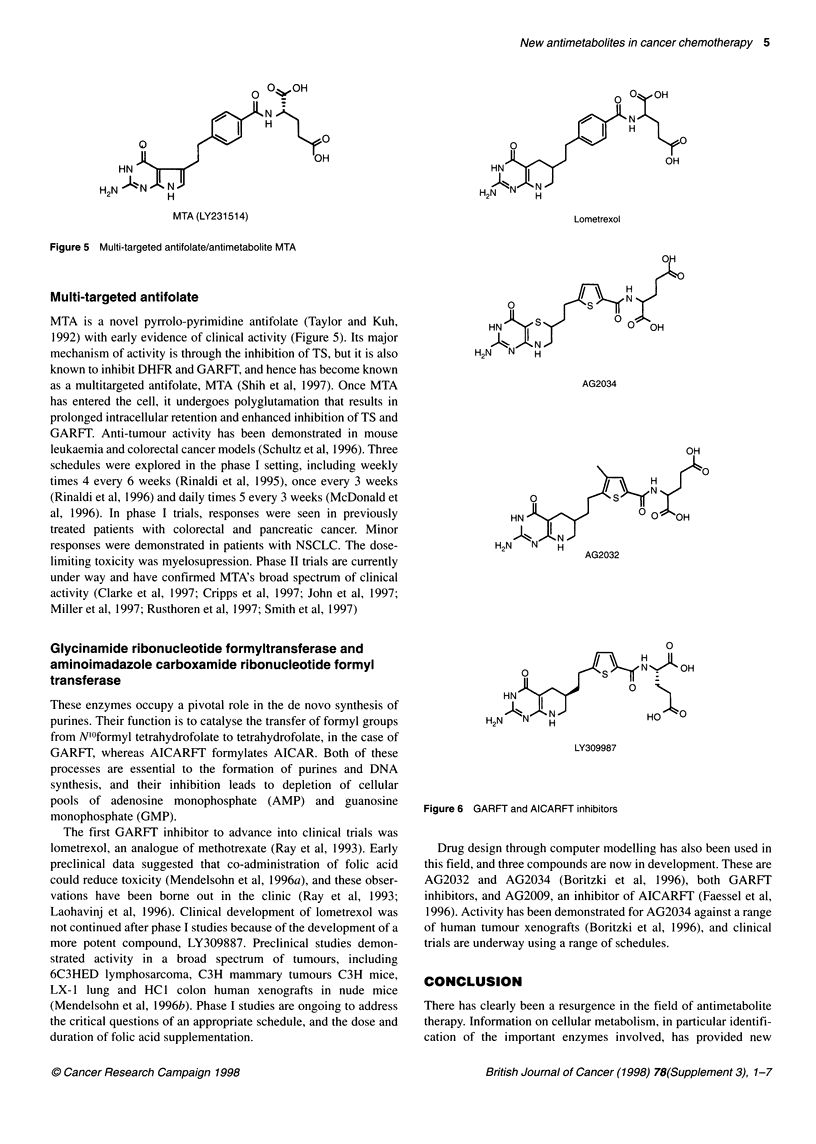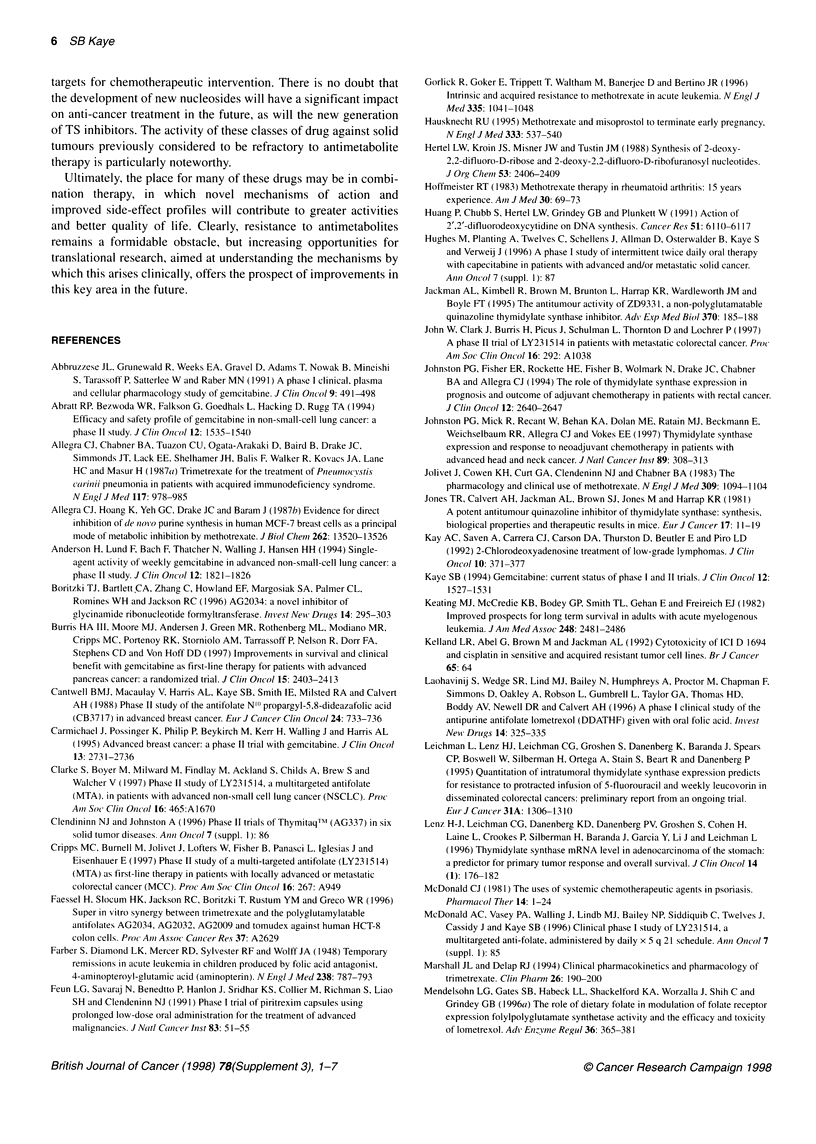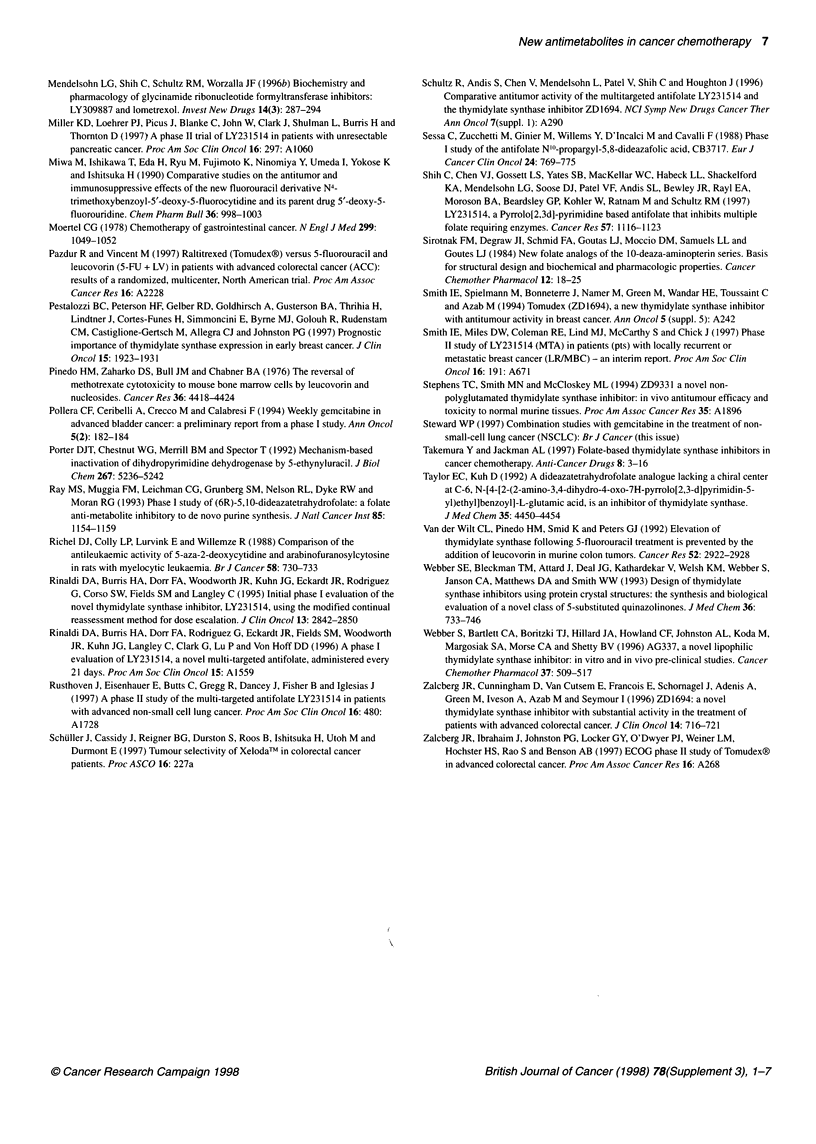# New antimetabolites in cancer chemotherapy and their clinical impact.

**DOI:** 10.1038/bjc.1998.747

**Published:** 1998

**Authors:** S. B. Kaye

**Affiliations:** Cancer Research Campaign Department of Medical Oncology, Beatson Oncology Centre, Western Infirmary, Glasgow, UK.

## Abstract

It is almost 50 years since antimetabolites were first found to have clinical antitumour activity, with Farber's discovery that aminopterin could cause remission in acute leukaemia. In the following 10 years, methotrexate, 6-mercaptopurine and 5-fluorouracil (5-FU) found their way into clinical practice. Subsequently, cytosine arabinoside was found to have activity in acute leukaemia, but, until recently, other significant developments have involved optimizing the efficacy of existing antimetabolites, including the use of leucovorin with methotrexate or 5-FU. Recently, new antimetabolites have become a fertile area for anti-cancer drug research. Gemcitabine (GEMZAR) has emerged as an important new agent in several tumour types, including pancreatic, non-small-cell lung, bladder, breast and ovarian cancers. Capecitabine is an intriguing new prodrug, offering tumour selectivity and prolonged tumour exposure to 5-FU. More potent thymidylate synthase inhibitors have also emerged; raltitrexed is now commercially available for the treatment of colorectal cancer. Others under development include LY231514, which has other sites of action, hence the acronym MTA (multi-targeted antifolate). A novel target is glycinamide ribonucleotide formyltransferase (GARFT) and LY309887 and AG2034 are undergoing clinical investigation as GARFT inhibitors. A critical element with LY309887 appears to be co-administration of folate. It seems entirely possible that several novel antimetabolites will establish themselves in clinical practice in future for the treatment of solid tumours.


British Journal of Cancer (1998) 78(Supplement 3), 1–7
© 1998 Cancer Research Campaign

### New antimetabolites in cancer chemotherapy and their clinical impact

**SB Kaye**

Cancer Research Campaign Department of Medical Oncology, Beatson Oncology Centre, Western Infirmary, Dumbarton Road, Glasgow G11 6NT, UK

**Summary** It is almost 50 years since antimetabolites were first found to have clinical antitumour activity, with Farber's discovery that aminopterin could cause remission in acute leukaemia. In the following 10 years, methotrexate, 6-mercaptopurine and 5-fluorouracil (5-FU) found their way into clinical practice. Subsequently, cytosine arabinoside was found to have activity in acute leukaemia, but, until recently, other significant developments have involved optimizing the efficacy of existing antimetabolites, including the use of leucovorin with methotrexate or 5-FU. Recently, new antimetabolites have become a fertile area for anti-cancer drug research. Gemcitabine (GEMZAR®) has emerged as an important new agent in several tumour types, including pancreatic, non-small-cell lung, bladder, breast and ovarian cancers. Capecitabine is an intriguing new prodrug, offering tumour selectivity and prolonged tumour exposure to 5-FU. More potent thymidylate synthase inhibitors have also emerged; raltitrexed is now commercially available for the treatment of colorectal cancer. Others under development include LY231514, which has other sites of action, hence the acronym MTA (multi-targeted antifolate). A novel target is glycinamide ribonucleotide formyltransferase (GARFT) and LY309887 and AG2034 are undergoing clinical investigation as GARFT inhibitors. A critical element with LY309887 appears to be co-administration of folate. It seems entirely possible that several novel antimetabolites will establish themselves in clinical practice in future for the treatment of solid tumours.

**Keywords:** antimetabolite; cancer chemotherapy; gemcitabine; multi-targeted antifolate; raltitrexed

Antimetabolites have been in use for the treatment of malignant disease for 50 years, since the discovery by Farber that aminopterin could cause remission of leukaemia (Farber et al, 1948). Since then, antimetabolites have been discovered that have found use in a variety of diseases other than cancer. For example, methotrexate is used in the treatment of psoriasis (McDonald, 1981) and rheumatoid arthritis (Hoffmeister, 1983), whereas trimetrexate has been used to treat *Pneumocystis carinii* infections in patients with acquired immune deficiency syndrome (Allegra et al, 1987*a*). But cancer therapy is their main application and for many years, 5-fluorouracil (5-FU) and methotrexate have been the mainstay of antimetabolite treatment in solid tumours. In recent years, however, several new antimetabolites have emerged in cancer treatment and these have provided the basis for further research.

An antimetabolite is defined as a drug that interferes with the normal metabolic processes within cells. Knowledge of these processes at a cellular level has increased in recent years, leading to the identification of a number of potential new targets. The metabolic processes of the cell are complex and involve many enzymes. Two important pathways exist, which give rise to the synthesis of purines and pyrimidines. Folate-derived co-factors are also involved in these processes as the one-carbon fragments provided by folates are essential to certain transformations, such as the conversion of deoxyuridine monophosphate (dUMP) to deoxythymidine monophosphate (dTMP). Inhibitors of vital enzymes in these pathways are being studied, including dihydrofolate reductase (DHFR), thymidylate synthase (TS), and glycinamide ribonucleotide formyltransferase (GARFT). These are illustrated in Figure 1, and will be discussed later. Other pharmacological targets have included the transport mechanisms responsible for ensuring drug uptake by the cell and, to this end, drugs have been developed that are better substrates for the reduced folate carrier (RFC) and the membrane-associated folate-binding protein (mFBP). These too will be discussed, along with compounds that have been designed to bypass these transport mechanisms. Nucleoside analogues, which play their role after incorporation into DNA and RNA, giving rise to chain termination and cell death or stasis, are among the most widely used of antimetabolites and will be reviewed in some detail. However, a full and complete discussion of every antimetabolite known would be beyond the scope of this review; therefore, only those examples that are of greatest interest will be discussed.

*Correspondence to:* SB Kaye

### POTENTIAL TARGETS FOR CHEMOTHERAPEUTIC INTERVENTION

### Dihydrofolate reductase inhibitors

Methotrexate (Figure 2), one of the earliest antimetabolites discovered, has been in use in cancer chemotherapy for over 30 years (Jolivet et al, 1983). It is an inhibitor of DHFR, which occupies a central position in the metabolic pathway. DHFR is responsible for the conversion of dihydrofolate to tetrahydrofolate and ultimately to 10-formyl tetrahydrofolate. The last compound provides the formyl group for glycinamide ribonucleotide formyltransferase (GARFT) and aminoimidazole carboxamide ribonucleotide formyl transferase (AICARFT). Thus, the inhibition of DHFR results in depletion of intracellular pools of reduced folates and ultimately in reduced synthesis of purines and pyrimidines.

**Figure 1** Folate biosynthetase pathways and inhibitors. Schematic representation of folate biosynthetic pathways and the enzymes involved. mFBP, membrane-associated folate binding protein; RFC, reduced folate carrier; FPGS, folylpolyglutamate synthase; dUMP, deoxyuridine monophosphate; $FH_4$, tetrahydrofolate; GAR, glycinamide ribonucleotide; TMP, thymidine monophosphate; $FH_2$, dihydrofolate; TS, thymidylate synthase; DHFR, dihydrofolate reductase; $Glu_n$, polyglutamate; dAMP, deoxyadenosine monophosphate; dGMP, deoxyguanosine monophosphate; TTP, thymidine triphosphate

Methotrexate

Edatrexate

Trimetrexate

Piritrexim

**Figure 2** DHFR inhibitors

The predominant mechanism of action of methotrexate is uncertain, as polyglutamated forms of the drug also inhibit TS and AICARFT (Allegra et al, 1987*b*).

Resistance to methotrexate arises by a variety of mechanisms, including impaired transport via the reduced folate carrier (Gorlick et al, 1996). This has inspired the search for other DHFR inhibitors, and led to the discovery of several methotrexate analogues including trimetrexate (Marshall and Delap, 1994) and edatrexate (Sirotnak et al, 1984). Trimetrexate is more lipophilic than methotrexate and is not dependent on the RFC for entry into the cell. This leads to higher concentrations of trimetrexate within the cell, although the drug does not undergo polyglutamylation. Clinical trials with trimetrexate are continuing; as yet there is no convincing evidence of superiority over methotrexate. Another DHFR inhibitor that has shown activity in humans is piritrexim. This is another compound that does not rely on the RFC, but enters the cell by means of passive diffusion. It has oral bioavailability of 75%, and clinical schedules with repeated low doses have been developed because of a relatively short half-life (3–5 h) (Feun et al, 1991). Activity has been seen for this drug in phase II trials in urothelial and head and neck tumours, as well as in melanoma, and evaluation is continuing.

Side-effects seen with this class of drug, as with most antimetabolites, consist mainly of toxicity to rapidly dividing cells, therefore mucositis, myelosuppression and thrombocytopenia are common. These effects are usually reversible and chemotherapy can be continued once levels have returned to normal. It has been known for several years that co-administration of leucovorin can reduce these toxicities to acceptable levels (Pinedo et al, 1976). Folic acid antagonists in general are embryotoxic and have caused spontaneous abortion in animals (Hausknecht, 1995).

### Nucleoside analogues

Another drug which has been in use for several decades is 5-fluorouracil (5-FU, Figure 3) (Moertel, 1978). It is a fluoropyrimidine, and is a member of the class of agents known as nucleoside analogues (Figure 3). In general, these agents function by replacing nucleosides in one or more normal cell functions because of their similarity to the naturally occurring substrates. They may fall into one of two main classes, either being incorporated into DNA and RNA synthesis or being responsible for inhibition of one of the enzymes essential to cell metabolism. The precise mechanism of action of 5-FU is unclear, and is partly a function of dose and schedule. However, it is likely that thymidylate synthase is the main target for the nucleoside of 5-FU, which binds to the active site of the enzyme in a similar manner to dUMP. This is followed by incorporation of the folate co-factor

© Cancer Research Campaign 1998

5-Fluorouracil

Capecitabine

Gemcitabine

**Figure 3** Nucleoside analogues

5, 10-methylenetetrahydrofolate that, combined with the fluorinated pyrimidine, locks the enzyme into an inhibited conformation resembling the transition state formed in the process of conversion of dUMP to thymidine by TS. Cellular levels of thymine are thus depleted and the enzyme is subsequently unable to function normally.

Given the importance of TS as a target for 5-FU, it would be of considerable clinical interest to establish whether prior knowledge of TS levels in tumour biopsies could predict response to 5-FU-based chemotherapy. It has been shown in several cell lines that lower levels of TS are associated with increased sensitivity to 5-FU (Van der Wilt et al, 1992). Clinical studies have now been reported in a number of tumour types.

First, and independent of drug response, clear correlations have been drawn between high TS levels and a poor prognosis. This may reflect an association with increased rates of tumour cell proliferation, or with post-transcriptional regulatory functions of TS protein. Large-scale studies in early-stage breast cancer (Pestalozzi et al, 1997), gastric cancer (Lenz et al, 1995) and primary rectal cancer (Johnston et al, 1994) all reach similar conclusions, with TS expression being measured either immunohistochemically, or using PCR to measure genetic expression of TS mRNA.

Second, correlations have also been drawn with response to 5-FU-based chemotherapy, in these and other studies. In studies in gastric cancer (Lenz et al, 1995) and in head and neck cancer (Johnston et al, 1997), 65 and 70 patients, respectively, received 5-FU-based neoadjuvant chemotherapy, and in both cases there was a significant ($P < 0.001$, $P = 0.02$, respectively) inverse correlation between TS expression level and response. A similar conclusion was drawn in a smaller study of 22 patients with advanced colorectal cancer (Leichman et al, 1995), in which a significant association ($P = 0.004$) between high TS expression and lack of response to infusional 5-FU was noted.

Conversely, in those studies that have attempted to correlate TS expression level with outcome of adjuvant chemotherapy; the opposite has been seen, i.e. patients with high TS levels have a better outcome with that therapy. This has been seen both in 278 node-positive breast cancer patients receiving CMF (cyclophosphamide, methotrexate, 5-FU) (Pestalozzi et al, 1997) and in 194 patients with Dukes' B and C rectal cancer receiving MOF (methyl CCNU, 5-FU vincristine) chemotherapy (Johnston et al, 1994). The reasons for the apparent paradox are not clear, but the data reported to date would certainly justify further translational studies to clarify the predictive role of TS expression; it remains quite possible that this will vary according to the type of tumour being treated.

Other recently developed nucleoside analogues include the 5-FU prodrug capecitabine (Figure 3) (Miwa et al, 1990). This cytidine analogue is administered as an oral formulation and passes unchanged through the intestinal mucosa. It is activated through a series of enzymatic steps in the liver and in tumour cells, with conversion to 5-FU in a potentially tumour-selective manner by the enzyme thymidine phosphorylase. Phase I studies of oral therapy with capecitabine in adult patients with a variety of advanced and/or metastatic solid cancers have shown that the drug is well tolerated (Hughes et al, 1996). Dose-limiting toxicities (DLT) included nausea, mucositis, diarrhoea and neutropenia. Palmar-plantar dyserythrodysthesia has also been seen. Pharmacological data indicate that to an extent, capecitabine simulates an i.v. protracted infusion of 5-FU; there is also clinical evidence of selective uptake in tumour biopsies after drug administration (Schüller et al, 1997). Evidence of response was seen in a variety of tumours, including breast, colorectal and oesophageal cancers, and occurred over a wide range of doses. Phase II trials have confirmed activity in breast and colorectal cancer, and randomized phase III studies are in progress.

Deoxynucleoside analogues are also finding an expanding application in cancer chemotherapy. The first of these agents was cytosine arabinoside (cytarabine). This is commonly used in the treatment of acute myeloblastic leukaemia (Keating et al, 1982) but has no significant action in solid tumours. After activation to the triphosphate, cytarabine has a range of modes of action, including incorporation into DNA and subsequent chain termination, stimulation of apoptosis and inhibition of DNA polymerase. Side-effects of treatment with cytarabine can include pancytopenia, alopecia, nausea, vomiting, fever, myalgia and bone and chest pain, and the drug is clearly schedule dependent in terms of toxicity and efficacy.

Analogues of cytarabine have been developed more recently, with broader spectrum anti-tumour activity. The first is gemcitabine, a difluorinated analogue (Hertel et al, 1988). Its activity is dependent upon the formation of a mononucleotide that is subsequently incorporated into DNA (Huang et al, 1991). One more residue is incorporated into the chain before chain termination takes place. This mechanism, termed 'masked chain termination', makes the recognition and excision of the modified DNA very difficult, and may partly explain the drug's broad activity. In addition, gemcitabine is capable of 'self-potentiation', whereby accumulation of the active metabolite leads to increased efficacy through reduction of intracellular pools of dCTP, after inhibition of ribonucleotide reductase, deoxycytidine deaminase, DNA polymerase and CTP synthetase.

© Cancer Research Campaign 1998

Clinical trials have been carried out to investigate the effect of schedule on the anti-tumour activity of gemcitabine, with the result that a weekly schedule was identified as having maximum potential (Abbruzzese et al, 1991). Preclinical studies have indicated that, unlike cytarabine, gemcitabine had a high level of activity in solid tumours, and this has been borne out in clinical trials. Activity has been seen for this agent in a variety of tumours, including pancreas, ovary, bladder, breast and lung cancers (Kaye, 1994; Abratt et al, 1995; Anderson et al, 1995; Carmichael et al, 1995; Burris et al, 1997). Improvements have been seen in the quality of life of patients with pancreatic cancer (Burris et al, 1997) and other trials have investigated the effect of gemcitabine in combination therapy, for example with cisplatin in the treatment of NSCLC (Steward, 1997).

Other agents in this class include ethynyl uracil (Porter et al, 1992), decitabine (Richel et al, 1988) and the fludarabine analogue cladribine (Kay et al, 1992), all of which are currently under clinical investigation.

### Thymidylate synthase inhibitors

This class of compound (Figure 4) has been the subject of intense research activity in recent years (Takemura and Jackman, 1997). Thymidylate synthase is essential to the synthesis of deoxythymidine monophosphate (dTMP) from deoxyuridine monophosphate (dUMP). This process is the sole source of dTMP in the cell and its inhibition leads to the depletion of cellular thymidine.

CB3717 was the first quinazoline TS inhibitor to undergo preclinical and clinical investigation. The compound was found to have excellent in vitro and in vivo anti-tumour activity (Jones et al, 1981). It enters the cell by means of the reduced folate carrier and requires polyglutamation for its activity. In phase I studies, responses were noted in lung and breast cancers, although renal toxicity was seen to be unpredictable and severe (Sessa et al, 1988). Subsequent phase II trials confirmed this and the development of the drug was discontinued (Cantwell et al, 1988). ZD1694 (raltitrexed) is a water-soluble analogue of CB3717 that was developed with the aim of reducing the side-effects seen in the parent compound (Kelland et al, 1992). This appeared to be successful, in that the renal toxicity observed with CB3717 was absent in raltitrexed, whereas the anti-tumour activity was retained. Clinical trials have confirmed the activity of raltitrexed in several tumour types, including breast and colorectal cancers (Zalcberg et al, 1996; Smith et al, 1994). Side-effects include neutropenia, transient elevations in transaminase levels, and gastrointestinal disturbances. Randomized trials in colorectal cancer have confirmed the activity of raltitrexed in comparison to standard 5-FU schedules, although the level of activity has been variable between studies (Pazdur and Vincent, 1997; Zalczberg, 1997). Raltitrexed was approved in the UK in March 1996 for use in the treatment of colorectal cancer.

A potential successor to raltitrexed is ZD9331. It, too, is a water-soluble compound but is not a substrate for folylpolyglutamyl synthetase (FPGS); hence it may overcome resistance to conventional polyglutamated TS inhibitors that may be due to defective FPGS activity (Stephens et al, 1994). Preclinical studies have shown impressive in vivo activity against human tumour xenografts, as well as activity in raltitrexed-resistant cell lines (Jackman et al, 1995). Phase I clinical trials with this compound are now underway.

CB3717

Raltitrexed

ZD9331

AG337

AG331

**Figure 4** Thymidylate synthase inhibitors

In recent years, medicinal chemists have used computer-aided molecular design for the development of new and more potent TS inhibitors. One example that has undergone extensive testing is AG337 (Thymitaq) (Webber et al, 1993). The compound is lipophilic and requires neither the RFC nor FPGS for activity, entering the cell by passive diffusion. Preclinical studies have shown activity in a range of human xenografts, though this is clearly schedule dependent (Webber et al, 1996). Phase I studies with AG337 have been conducted with a range of oral and i.v. schedules. Toxicities seen include nausea, myelosuppression and mucositis. Activity has been seen in head and neck cancer, pancreatic cancer and hepatoma (Clendininn and Johnston, 1996), and randomized trials, using a protracted i.v. infusion of AG337, are underway.

 *© Cancer Research Campaign 1998*

MTA (LY231514)

**Figure 5** Multi-targeted antifolate/antimetabolite MTA

### Multi-targeted antifolate

MTA is a novel pyrrolo-pyrimidine antifolate (Taylor and Kuh, 1992) with early evidence of clinical activity (Figure 5). Its major mechanism of activity is through the inhibition of TS, but it is also known to inhibit DHFR and GARFT, and hence has become known as a multitargeted antifolate, MTA (Shih et al, 1997). Once MTA has entered the cell, it undergoes polyglutamation that results in prolonged intracellular retention and enhanced inhibition of TS and GARFT. Anti-tumour activity has been demonstrated in mouse leukaemia and colorectal cancer models (Schultz et al, 1996). Three schedules were explored in the phase I setting, including weekly times 4 every 6 weeks (Rinaldi et al, 1995), once every 3 weeks (Rinaldi et al, 1996) and daily times 5 every 3 weeks (McDonald et al, 1996). In phase I trials, responses were seen in previously treated patients with colorectal and pancreatic cancer. Minor responses were demonstrated in patients with NSCLC. The dose-limiting toxicity was myelosupression. Phase II trials are currently under way and have confirmed MTA's broad spectrum of clinical activity (Clarke et al, 1997; Cripps et al, 1997; John et al, 1997; Miller et al, 1997; Rusthoren et al, 1997; Smith et al, 1997)

### Glycinamide ribonucleotide formyltransferase and aminoimadazole carboxamide ribonucleotide formyl transferase

These enzymes occupy a pivotal role in the de novo synthesis of purines. Their function is to catalyse the transfer of formyl groups from $N^{10}$formyl tetrahydrofolate to tetrahydrofolate, in the case of GARFT, whereas AICARFT formylates AICAR. Both of these processes are essential to the formation of purines and DNA synthesis, and their inhibition leads to depletion of cellular pools of adenosine monophosphate (AMP) and guanosine monophosphate (GMP).

The first GARFT inhibitor to advance into clinical trials was lometrexol, an analogue of methotrexate (Ray et al, 1993). Early preclinical data suggested that co-administration of folic acid could reduce toxicity (Mendelsohn et al, 1996*a*), and these observations have been borne out in the clinic (Ray et al, 1993; Laohavinj et al, 1996). Clinical development of lometrexol was not continued after phase I studies because of the development of a more potent compound, LY309887. Preclinical studies demonstrated activity in a broad spectrum of tumours, including 6C3HED lymphosarcoma, C3H mammary tumours C3H mice, LX-1 lung and HC1 colon human xenografts in nude mice (Mendelsohn et al, 1996*b*). Phase I studies are ongoing to address the critical questions of an appropriate schedule, and the dose and duration of folic acid supplementation.

Lometrexol

AG2034

AG2032

LY309987

**Figure 6** GARFT and AICARFT inhibitors

Drug design through computer modelling has also been used in this field, and three compounds are now in development. These are AG2032 and AG2034 (Boritzki et al, 1996), both GARFT inhibitors, and AG2009, an inhibitor of AICARFT (Faessel et al, 1996). Activity has been demonstrated for AG2034 against a range of human tumour xenografts (Boritzki et al, 1996), and clinical trials are underway using a range of schedules.

### CONCLUSION

There has clearly been a resurgence in the field of antimetabolite therapy. Information on cellular metabolism, in particular identification of the important enzymes involved, has provided new

© Cancer Research Campaign 1998

targets for chemotherapeutic intervention. There is no doubt that the development of new nucleosides will have a significant impact on anti-cancer treatment in the future, as will the new generation of TS inhibitors. The activity of these classes of drug against solid tumours previously considered to be refractory to antimetabolite therapy is particularly noteworthy.

Ultimately, the place for many of these drugs may be in combination therapy, in which novel mechanisms of action and improved side-effect profiles will contribute to greater activities and better quality of life. Clearly, resistance to antimetabolites remains a formidable obstacle, but increasing opportunities for translational research, aimed at understanding the mechanisms by which this arises clinically, offers the prospect of improvements in this key area in the future.

### REFERENCES

Abbruzzese JL, Grunewald R, Weeks EA, Gravel D, Adams T, Nowak B, Mineishi S, Tarassoff P, Satterlee W and Raber MN (1991) A phase I clinical, plasma and cellular pharmacology study of gemcitabine. *J Clin Oncol* **9**: 491–498

Abratt RP, Bezwoda WR, Falkson G, Goedhals L, Hacking D, Rugg TA (1994) Efficacy and safety profile of gemcitabine in non-small-cell lung cancer: a phase II study. *J Clin Oncol* **12**: 1535–1540

Allegra CJ, Chabner BA, Tuazon CU, Ogata-Arakaki D, Baird B, Drake JC, Simmonds JT, Lack EE, Shelhamer JH, Balis F, Walker R, Kovacs JA, Lane HC and Masur H (1987*a*) Trimetrexate for the treatment of *Pneumocystis carinii* pneumonia in patients with acquired immunodeficiency syndrome. *N Engl J Med* **117**: 978–985

Allegra CJ, Hoang K, Yeh GC, Drake JC and Baram J (1987*b*) Evidence for direct inhibition of *de novo* purine synthesis in human MCF-7 breast cells as a principal mode of metabolic inhibition by methotrexate. *J Biol Chem* **262**: 13520–13526

Anderson H, Lund F, Bach F, Thatcher N, Walling J, Hansen HH (1994) Single-agent activity of weekly gemcitabine in advanced non-small-cell lung cancer: a phase II study. *J Clin Oncol* **12**: 1821–1826

Boritzki TJ, Bartlett CA, Zhang C, Howland EF, Margosiak SA, Palmer CL, Romines WH and Jackson RC (1996) AG2034: a novel inhibitor of glycinamide ribonucleotide formyltransferase. *Invest New Drugs* **14**: 295–303

Burris HA III, Moore MJ, Andersen J, Green MR, Rothenberg ML, Modiano MR, Cripps MC, Portenoy RK, Storniolo AM, Tarrassoff P, Nelson R, Dorr FA, Stephens CD and Von Hoff DD (1997) Improvements in survival and clinical benefit with gemcitabine as first-line therapy for patients with advanced pancreas cancer: a randomized trial. *J Clin Oncol* **15**: 2403–2413

Cantwell BMJ, Macaulay V, Harris AL, Kaye SB, Smith IE, Milsted RA and Calvert AH (1988) Phase II study of the antifolate $N^{10}$ propargyl-5,8-dideazafolic acid (CB3717) in advanced breast cancer. *Eur J Cancer Clin Oncol* **24**: 733–736

Carmichael J, Possinger K, Philip P, Beykirch M, Kerr H, Walling J and Harris AL (1995) Advanced breast cancer: a phase II trial with gemcitabine. *J Clin Oncol* **13**: 2731–2736

Clarke S, Boyer M, Milward M, Findlay M, Ackland S, Childs A, Brew S and Walcher V (1997) Phase II study of LY231514, a multitargeted antifolate (MTA), in patients with advanced non-small cell lung cancer (NSCLC). *Proc Am Soc Clin Oncol* **16**: 465:A1670

Clendininn NJ and Johnston A (1996) Phase II trials of Thymitaq™ (AG337) in six solid tumor diseases. *Ann Oncol* **7** (suppl. 1): 86

Cripps MC, Burnell M, Jolivet J, Lofters W, Fisher B, Panasci L, Iglesias J and Eisenhauer E (1997) Phase II study of a multi-targeted antifolate (LY231514) (MTA) as first-line therapy in patients with locally advanced or metastatic colorectal cancer (MCC). *Proc Am Soc Clin Oncol* **16**: 267: A949

Faessel H, Slocum HK, Jackson RC, Boritzki T, Rustum YM and Greco WR (1996) Super in vitro synergy between trimetrexate and the polyglutamylatable antifolates AG2034, AG2032, AG2009 and tomudex against human HCT-8 colon cells. *Proc Am Assoc Cancer Res* **37**: A2629

Farber S, Diamond LK, Mercer RD, Sylvester RF and Wolff JA (1948) Temporary remissions in acute leukemia in children produced by folic acid antagonist, 4-aminopteroyl-glutamic acid (aminopterin). *N Engl J Med* **238**: 787–793

Feun LG, Savaraj N, Benedtto P, Hanlon J, Sridhar KS, Collier M, Richman S, Liao SH and Clendeninn NJ (1991) Phase I trial of piritrexim capsules using prolonged low-dose oral administration for the treatment of advanced malignancies. *J Natl Cancer Inst* **83**: 51–55

Gorlick R, Goker E, Trippett T, Waltham M, Banerjee D and Bertino JR (1996) Intrinsic and acquired resistance to methotrexate in acute leukemia. *N Engl J Med* **335**: 1041–1048

Hausknecht RU (1995) Methotrexate and misoprostol to terminate early pregnancy. *N Engl J Med* **333**: 537–540

Hertel LW, Kroin JS, Misner JW and Tustin JM (1988) Synthesis of 2-deoxy-2,2-difluoro-D-ribose and 2-deoxy-2,2-difluoro-D-ribofuranosyl nucleotides. *J Org Chem* **53**: 2406–2409

Hoffmeister RT (1983) Methotrexate therapy in rheumatoid arthritis: 15 years experience. *Am J Med* **30**: 69–73

Huang P, Chubb S, Hertel LW, Grindey GB and Plunkett W (1991) Action of 2′,2′-difluorodeoxycytidine on DNA synthesis. *Cancer Res* **51**: 6110–6117

Hughes M, Planting A, Twelves C, Schellens J, Allman D, Osterwalder B, Kaye S and Verweij J (1996) A phase I study of intermittent twice daily oral therapy with capecitabine in patients with advanced and/or metastatic solid cancer. *Ann Oncol* 7 (suppl. 1): 87

Jackman AL, Kimbell R, Brown M, Brunton L, Harrap KR, Wardleworth JM and Boyle FT (1995) The antitumour activity of ZD9331, a non-polyglutamatable quinazoline thymidylate synthase inhibitor. *Adv Exp Med Biol* **370**: 185–188

John W, Clark J, Burris H, Picus J, Schulman L, Thornton D and Lochrer P (1997) A phase II trial of LY231514 in patients with metastatic colorectal cancer. *Proc Am Soc Clin Oncol* **16**: 292: A1038

Johnston PG, Fisher ER, Rockette HE, Fisher B, Wolmark N, Drake JC, Chabner BA and Allegra CJ (1994) The role of thymidylate synthase expression in prognosis and outcome of adjuvant chemotherapy in patients with rectal cancer. *J Clin Oncol* **12**: 2640–2647

Johnston PG, Mick R, Recant W, Behan KA, Dolan ME, Ratain MJ, Beckmann E, Weichselbaum RR, Allegra CJ and Vokes EE (1997) Thymidylate synthase expression and response to neoadjuvant chemotherapy in patients with advanced head and neck cancer. *J Natl Cancer Inst* **89**: 308–313

Jolivet J, Cowen KH, Curt GA, Clendeninn NJ and Chabner BA (1983) The pharmacology and clinical use of methotrexate. *N Engl J Med* **309**: 1094–1104

Jones TR, Calvert AH, Jackman AL, Brown SJ, Jones M and Harrap KR (1981) A potent antitumour quinazoline inhibitor of thymidylate synthase: synthesis, biological properties and therapeutic results in mice. *Eur J Cancer* **17**: 11–19

Kay AC, Saven A, Carrera CJ, Carson DA, Thurston D, Beutler E and Piro LD (1992) 2-Chlorodeoxyadenosine treatment of low-grade lymphomas. *J Clin Oncol* **10**: 371–377

Kaye SB (1994) Gemcitabine: current status of phase I and II trials. *J Clin Oncol* **12**: 1527–1531

Keating MJ, McCredie KB, Bodey GP, Smith TL, Gehan E and Freireich EJ (1982) Improved prospects for long term survival in adults with acute myelogenous leukemia. *J Am Med Assoc* **248**: 2481–2486

Kelland LR, Abel G, Brown M and Jackman AL (1992) Cytotoxicity of ICI D 1694 and cisplatin in sensitive and acquired resistant tumor cell lines. *Br J Cancer* **65**: 64

Laohavinij S, Wedge SR, Lind MJ, Bailey N, Humphreys A, Proctor M, Chapman F, Simmons D, Oakley A, Robson L, Gumbrell L, Taylor GA, Thomas HD, Boddy AV, Newell DR and Calvert AH (1996) A phase I clinical study of the antipurine antifolate lometrexol (DDATHF) given with oral folic acid. *Invest New Drugs* **14**: 325–335

Leichman L, Lenz HJ, Leichman CG, Groshen S, Danenberg K, Baranda J, Spears CP, Boswell W, Silberman H, Ortega A, Stain S, Beart R and Danenberg P (1995) Quantitation of intratumoral thymidylate synthase expression predicts for resistance to protracted infusion of 5-fluorouracil and weekly leucovorin in disseminated colorectal cancers: preliminary report from an ongoing trial. *Eur J Cancer* **31A**: 1306–1310

Lenz H-J, Leichman CG, Danenberg KD, Danenberg PV, Groshen S, Cohen H, Laine L, Crookes P, Silberman H, Baranda J, Garcia Y, Li J and Leichman L (1996) Thymidylate synthase mRNA level in adenocarcinoma of the stomach: a predictor for primary tumor response and overall survival. *J Clin Oncol* **14** **(1)**: 176–182

McDonald CJ (1981) The uses of systemic chemotherapeutic agents in psoriasis. *Pharmacol Ther* **14**: 1–24

McDonald AC, Vasey PA, Walling J, Lindb MJ, Bailey NP, Siddiquib C, Twelves J, Cassidy J and Kaye SB (1996) Clinical phase I study of LY231514, a multitargeted anti-folate, administered by daily × 5 q 21 schedule. *Ann Oncol* **7** (suppl. 1): 85

Marshall JL and Delap RJ (1994) Clinical pharmacokinetics and pharmacology of trimetrexate. *Clin Pharm* **26**: 190–200

Mendelsohn LG, Gates SB, Habeck LL, Shackelford KA, Worzalla J, Shih C and Grindey GB (1996*a*) The role of dietary folate in modulation of folate receptor expression folylpolyglutamate synthetase activity and the efficacy and toxicity of lometrexol. *Adv Enzyme Regul* **36**: 365–381

© Cancer Research Campaign 1998

Mendelsohn LG, Shih C, Schultz RM, Worzalla JF (1996*b*) Biochemistry and pharmacology of glycinamide ribonucleotide formyltransferase inhibitors: LY309887 and lometrexol. *Invest New Drugs* **14(3)**: 287–294

Miller KD, Loehrer PJ, Picus J, Blanke C, John W, Clark J, Shulman L, Burris H and Thornton D (1997) A phase II trial of LY231514 in patients with unresectable pancreatic cancer. *Proc Am Soc Clin Oncol* **16**: 297: A1060

Miwa M, Ishikawa T, Eda H, Ryu M, Fujimoto K, Ninomiya Y, Umeda I, Yokose K and Ishitsuka H (1990) Comparative studies on the antitumor and immunosuppressive effects of the new fluorouracil derivative $N^4$-trimethoxybenzoyl-5′-deoxy-5-fluorocytidine and its parent drug 5′-deoxy-5-fluorouridine. *Chem Pharm Bull* **36**: 998–1003

Moertel CG (1978) Chemotherapy of gastrointestinal cancer. *N Engl J Med* **299**: 1049–1052

Pazdur R and Vincent M (1997) Raltitrexed (Tomudex®) versus 5-fluorouracil and leucovorin (5-FU + LV) in patients with advanced colorectal cancer (ACC): results of a randomized, multicenter, North American trial. *Proc Am Assoc Cancer Res* **16**: A2228

Pestalozzi BC, Peterson HF, Gelber RD, Goldhirsch A, Gusterson BA, Thrihia H, Lindtner J, Cortes-Funes H, Simmoncini E, Byrne MJ, Golouh R, Rudenstam CM, Castiglione-Gertsch M, Allegra CJ and Johnston PG (1997) Prognostic importance of thymidylate synthase expression in early breast cancer. *J Clin Oncol* **15**: 1923–1931

Pinedo HM, Zaharko DS, Bull JM and Chabner BA (1976) The reversal of methotrexate cytotoxicity to mouse bone marrow cells by leucovorin and nucleosides. *Cancer Res* **36**: 4418–4424

Pollera CF, Ceribelli A, Crecco M and Calabresi F (1994) Weekly gemcitabine in advanced bladder cancer: a preliminary report from a phase I study. *Ann Oncol* **5(2)**: 182–184

Porter DJT, Chestnut WG, Merrill BM and Spector T (1992) Mechanism-based inactivation of dihydropyrimidine dehydrogenase by 5-ethynyluracil. *J Biol Chem* **267**: 5236–5242

Ray MS, Muggia FM, Leichman CG, Grunberg SM, Nelson RL, Dyke RW and Moran RG (1993) Phase I study of (6R)-5,10-dideazatetrahydrofolate: a folate anti-metabolite inhibitory to de novo purine synthesis. *J Natl Cancer Inst* **85**: 1154–1159

Richel DJ, Colly LP, Lurvink E and Willemze R (1988) Comparison of the antileukaemic activity of 5-aza-2-deoxycytidine and arabinofuranosylcytosine in rats with myelocytic leukaemia. *Br J Cancer* **58**: 730–733

Rinaldi DA, Burris HA, Dorr FA, Woodworth JR, Kuhn JG, Eckardt JR, Rodriguez G, Corso SW, Fields SM and Langley C (1995) Initial phase I evaluation of the novel thymidylate synthase inhibitor, LY231514, using the modified continual reassessment method for dose escalation. *J Clin Oncol* **13**: 2842–2850

Rinaldi DA, Burris HA, Dorr FA, Rodriguez G, Eckardt JR, Fields SM, Woodworth JR, Kuhn JG, Langley C, Clark G, Lu P and Von Hoff DD (1996) A phase I evaluation of LY231514, a novel multi-targeted antifolate, administered every 21 days. *Proc Am Soc Clin Oncol* **15**: A1559

Rusthoven J, Eisenhauer E, Butts C, Gregg R, Dancey J, Fisher B and Iglesias J (1997) A phase II study of the multi-targeted antifolate LY231514 in patients with advanced non-small cell lung cancer. *Proc Am Soc Clin Oncol* **16**: 480: A1728

Schüller J, Cassidy J, Reigner BG, Durston S, Roos B, Ishitsuka H, Utoh M and Durmont E (1997) Tumour selectivity of Xeloda™ in colorectal cancer patients. *Proc ASCO* **16**: 227a

Schultz R, Andis S, Chen V, Mendelsohn L, Patel V, Shih C and Houghton J (1996) Comparative antitumor activity of the multitargeted antifolate LY231514 and the thymidylate synthase inhibitor ZD1694. *NCI Symp New Drugs Cancer Ther Ann Oncol* **7**(suppl. 1): A290

Sessa C, Zucchetti M, Ginier M, Willems Y, D'Incalci M and Cavalli F (1988) Phase I study of the antifolate $N^{10}$-propargyl-5,8-dideazafolic acid, CB3717. *Eur J Cancer Clin Oncol* **24**: 769–775

Shih C, Chen VJ, Gossett LS, Yates SB, MacKellar WC, Habeck LL, Shackelford KA, Mendelsohn LG, Soose DJ, Patel VF, Andis SL, Bewley JR, Rayl EA, Moroson BA, Beardsley GP, Kohler W, Ratnam M and Schultz RM (1997) LY231514, a Pyrrolo[2,3d]-pyrimidine based antifolate that inhibits multiple folate requiring enzymes. *Cancer Res* **57**: 1116–1123

Sirotnak FM, Degraw JI, Schmid FA, Goutas LJ, Moccio DM, Samuels LL and Goutes LJ (1984) New folate analogs of the 10-deaza-aminopterin series. Basis for structural design and biochemical and pharmacologic properties. *Cancer Chemother Pharmacol* **12**: 18–25

Smith IE, Spielmann M, Bonneterre J, Namer M, Green M, Wandar HE, Toussaint C and Azab M (1994) Tomudex (ZD1694), a new thymidylate synthase inhibitor with antitumour activity in breast cancer. *Ann Oncol* **5** (suppl. 5): A242

Smith IE, Miles DW, Coleman RE, Lind MJ, McCarthy S and Chick J (1997) Phase II study of LY231514 (MTA) in patients (pts) with locally recurrent or metastatic breast cancer (LR/MBC) – an interim report. *Proc Am Soc Clin Oncol* **16**: 191: A671

Stephens TC, Smith MN and McCloskey ML (1994) ZD9331 a novel non-polyglutamated thymidylate synthase inhibitor: in vivo antitumour efficacy and toxicity to normal murine tissues. *Proc Am Assoc Cancer Res* **35**: A1896

Steward WP (1997) Combination studies with gemcitabine in the treatment of non-small-cell lung cancer (NSCLC): *Br J Cancer* (this issue)

Takemura Y and Jackman AL (1997) Folate-based thymidylate synthase inhibitors in cancer chemotherapy. *Anti-Cancer Drugs* **8**: 3–16

Taylor EC, Kuh D (1992) A dideazatetrahydrofolate analogue lacking a chiral center at C-6, N-[4-[2-(2-amino-3,4-dihydro-4-oxo-7H-pyrrolo[2,3-d]pyrimidin-5-yl)ethyl]benzoyl]-L-glutamic acid, is an inhibitor of thymidylate synthase. *J Med Chem* **35**: 4450–4454

Van der Wilt CL, Pinedo HM, Smid K and Peters GJ (1992) Elevation of thymidylate synthase following 5-fluorouracil treatment is prevented by the addition of leucovorin in murine colon tumors. *Cancer Res* **52**: 2922–2928

Webber SE, Bleckman TM, Attard J, Deal JG, Kathardekar V, Welsh KM, Webber S, Janson CA, Matthews DA and Smith WW (1993) Design of thymidylate synthase inhibitors using protein crystal structures: the synthesis and biological evaluation of a novel class of 5-substituted quinazolinones. *J Med Chem* **36**: 733–746

Webber S, Bartlett CA, Boritzki TJ, Hillard JA, Howland CF, Johnston AL, Koda M, Margosiak SA, Morse CA and Shetty BV (1996) AG337, a novel lipophilic thymidylate synthase inhibitor: in vitro and in vivo pre-clinical studies. *Cancer Chemother Pharmacol* **37**: 509–517

Zalcberg JR, Cunningham D, Van Cutsem E, Francois E, Schornagel J, Adenis A, Green M, Iveson A, Azab M and Seymour I (1996) ZD1694: a novel thymidylate synthase inhibitor with substantial activity in the treatment of patients with advanced colorectal cancer. *J Clin Oncol* **14**: 716–721

Zalcberg JR, Ibrahaim J, Johnston PG, Locker GY, O'Dwyer PJ, Weiner LM, Hochster HS, Rao S and Benson AB (1997) ECOG phase II study of Tomudex® in advanced colorectal cancer. *Proc Am Assoc Cancer Res* **16**: A268

© Cancer Research Campaign 1998